# The code of sustainable success in fitness apps: social comparison mechanism enabled by user facilitated supports

**DOI:** 10.3389/fpubh.2025.1632598

**Published:** 2025-08-04

**Authors:** Kuang Wu, Qi Li, Yang Liu

**Affiliations:** ^1^School of Economics and Finance, Xi’an Jiaotong University, Xi’an, China; ^2^School of Management Science and Information Engineering, Jilin University of Finance and Economics, Changchun, China

**Keywords:** sustainable success, fitness app, social comparison theory, social support theory, public health

## Abstract

**Introduction:**

Integrating social comparison theory and social support theory, this study develops a holistic model examining how fitness app social features influence sustainable development of users. This study not only extends the influence of user facilitated support, but also explores the social comparison factors, providing unique insights into interpreting the mechanism of continuous use intention and fitness intention.

**Methods:**

This study collects empirical data by distributing questionnaires. We use structural equation modeling to examine the relationship between fitness app social features, continuous use intention and fitness intention.

**Results:**

This study finds that information support, emotional support and upward comparison affect social presence and fitness interest positively, which in turn affect continuous use intention and fitness intention positively. Interestingly, downward comparison affects social presence negatively.

**Discussion:**

These findings provide references for fitness platform practitioners to design marketing strategies and thus improve the sustainable commercial value of fitness apps. In addition, the results can be applied to promote users’ fitness motivation and thus offer sustainable health value to the society.

## 1 Introduction

Ensuring that fitness apps deliver sustainable health, commercial, and social value is vital for maintaining users’ continuous engagement and motivation. Furthermore, the sustainability of fitness apps manifests in three principal dimensions. First, health is a continuous process of effort. If individuals use fitness apps continuously, they will achieve the expected fitness outcomes, and thus will be able to improve their health levels (1). Fitness apps provide users with universal health benefits, thus the fitness apps need to be used and developed sustainably. Second, fitness apps possess sustainable commercial value. Fitness apps integrate effective commercial model of electronic business and sustain a large user base. And commercial value of fitness apps is increasing through users’ sustained exercise and continuous use of fitness apps. Third, fitness apps provide users with sustainable social support, enabling them to engage in fitness training with a positive mindset, thereby contributing to the improvement of fitness outcomes (2). The social interaction features provided by fitness apps create sustainable social value for users. As well as fostering a social atmosphere that encourages sustainable development of fitness and motivates fitness participation of users.

Especially, with the continuous improvement of China’s overall economic level, the public is increasingly focusing on exercise and health, resulting in a growing demand for fitness. According to data from the General Administration of Sport of China, the top three fitness apps in terms of average monthly active users in the fourth quarter of 2022 were Keep (19.583 million), Xiaomi Sports (7.273 million), and Tangdou (6.965 million). In the first half of 2023, Keep’s average monthly subscription membership reached 3.02 million (3). According to a report by ReportLinker, fitness apps are expected to experience exponential growth globally in the coming years. The revenue generated by fitness apps will reach $56.29 billion by 2030 (4). It is widely acknowledged that fitness apps will receive broader attention in the future.

Fitness apps integrate mobile communication and multimedia technology within the realm of exercise and fitness, establishing a “terminal + platform + service” model. Fitness apps not only offer users fitness courses and record fitness data, but also include social features. While using the app, users can interact with others, such as sharing fitness data and status, liking and commenting on other users’ fitness posts, and receiving advice and emotional support. Furthermore, users can view their fitness performance rankings and compare themselves with others to evaluate their current fitness levels better. The extensive social features of fitness apps facilitate a more efficient and enjoyable engagement in fitness activities, thereby encouraging users to continue their usage. Current research primarily examines fitness apps from the perspectives of external variables (such as fitness motivation, perceived ease of use and perceived costs), transaction costs, and media dependency (5, 6), with relatively few studies investigating the relationship between the social features of fitness apps and users’ continuous use intention.

This article takes fitness apps as the research context and investigates the continuous use intention and fitness intention of users based on the SOR theory. Building on prior research, the social features of fitness apps are categorized into information support, emotional support, upward comparison, and downward comparison in this study. It is posited that these social features stimulate organism of users, thereby influencing their continuous use intention. In summary, this study asks: How do information support, emotional support, upward comparison, and downward comparison (the social features in fitness apps) influence users’ social presence and fitness interest, and in turn their continuous use intention and fitness intention? The findings of this study hold significant implications for fitness app practitioners in enhancing marketing strategies and improving the sustainable commercial value of fitness apps.

## 2 Literature review

### 2.1 Fitness apps

The demand for health and exercise among individuals is steadily increasing, and the number of users of fitness apps is also growing annually. Therefore, exploring the factors that influence people’s continuous use of fitness apps has become a significant issue for scholars in this field. Existing literature primarily investigates factors affecting usage attitudes, usage motivations, participation intentions, and continuous use intentions. For instance, Song et al. (7) conducted research based on affordance theory, revealing that social presence, immersion, and credibility positively influenced users’ continuous use intentions of fitness apps. Durau et al. (8) discovered that fitness attitudes and motivation impacted participants’ fitness intentions. Fitness apps offer users fitness videos and online communication services, enabling them to obtain fitness guidance, engage in fitness activities, post fitness updates, and like and comment on other users’ fitness updates. The process involves social interaction between users and the app, as well as among users. During this process, individuals receive not only information support but also emotional support. For example, when individuals receive positive comments from others, they will experience a sense of joy, which subsequently enhances their fitness motivation. Furthermore, through the social features of fitness apps, individuals can view their fitness rankings in comparison to others, leading to social comparison, which fosters a competitive environment that motivates people to engage in fitness exercises. Thus, it is evident that the social features of fitness apps play a crucial role in encouraging individuals to use the app more actively and participate in fitness activities. However, there has been limited research conducted from the perspective of social features. Thus, this paper aims to explore whether the two social features of fitness apps, including social support and social comparison, can influence individuals’ continuous use intentions of fitness apps.

### 2.2 Social support theory

Social support refers to the experience of individuals receiving respect, care, and assistance from others within their social networks. The traditional concept of social support primarily encompasses instrumental support, informational support, and emotional support (9). Social support theory is frequently applied in research on online user communities, occupational burnout, entrepreneurship, and other areas. For example, van Brakel et al. (10) studied the impact of social support on users of social VR platforms. Le et al. (11) based on the stimulus-organism-response (SOR) model, elucidated the mechanisms behind privacy disclosure behavior from the perspective of social support. In the context of fitness apps, Sun and Jiang (12) categorized social support into informational support, self-esteem support, and companionship support, with findings indicating that social support motivated users’ physical exercise through fitness apps. Huang et al. (13) also classified social support into three categories and examined its influence on user participation in physical exercise, concluding that social support could not explain participation in physical exercise. In fitness apps, social support is realized through interactions with the app interface and other users. For instance, users continuously receive informational support regarding exercises through fitness apps. Fitness apps also provide companionship and emotional support by displaying friends’ physical activities. Users’ fitness updates receive likes and comments from others, thereby fostering a sense of support. Research has found that users of fitness apps engage in exercise to receive praise from others (14), indicating that social support is an effective motivational function that fitness apps possess for users.

### 2.3 Social comparison theory

Social comparison theory was proposed by Festinger (15). The theory posits that individuals possess an intrinsic motivation to evaluate their self-concept and abilities. Driven by this motivation, individuals seek information for self-assessment. However, in the absence of objective and scientific evaluation methods, individuals resort to comparing their abilities with others for self-evaluation. Social comparison typically includes upward and downward comparisons, where individuals compare themselves with others who are in better or worse conditions across multiple dimensions such as abilities and achievements (16). The theory is frequently applied in research on social websites and online social games. For instance, Burke and Rains (17) studied the relationship between individuals’ exposure to others’ exercise posts on social websites and their concerns about weight and attitudes toward physical activity. Kashian and Liu (18) found fitness posts sharing successful experience on social media were positively correlated with self-efficacy. Esteves et al. (19) explored the impact of social comparison on players’ continuous gaming intentions. Huang et al. (13) discovered that upward comparison was positively correlated with participation in physical activities, while downward comparison was negatively correlated with it. However, some studies suggest that social comparison with ideal images of others can lower one’s self-evaluation of their appearance, leading to increased dissatisfaction with their body (20, 21). For example, Arroyo and Brunner (22) found that browsing friends’ fitness posts was positively correlated with negative body talk. Fitness apps typically record individuals’ exercise data and performance, ranking their athletic achievements against friends. Features such as performance rankings create a psychological environment for social comparison, aiding users in assessing their fitness levels accurately. As one of the unique social features of fitness apps, social comparison is significant and should be studied as a behavior change mechanism within the fitness environment (23). In the process of using fitness apps, whether upward comparison and downward comparison have a positive or negative impact on users also requires further research.

### 2.4 Stimulus-organism-response model

The Stimulus-organism-response model originates from the field of environmental psychology, where S represents various stimuli from the external environment. External stimuli (S) affect the internal states (O) of the organism, which in turn drives the subsequent behavioral response (R) (24). The SOR model is primarily used to study the internal state responses of individuals to various external environmental stimuli. And the behavioral responses resulting from changes in internal state responses. The organism refers to cognitive and emotional responses. Cognitive responses indicate the psychological processes involved in acquiring, retaining, retrieving, and processing information, while emotional responses reflect the arousal, resonance, and pleasure generated by environmental stimuli (25). Individuals transform environmental stimuli into meaningful information and influence their subsequent specific behavioral responses.

Scholars have utilized the SOR model to investigate individuals’ behavioral responses across various research domains, including impulse buying, online learning, and online social networks (26, 27). In the field of fitness, recent studies have also applied the SOR theory to investigate the impact of short video features and platform characteristics as environmental stimuli on individuals’ internal states. As well as the resulting behavioral responses related to fitness apps. For example, Chen et al. (28) found a positive correlation between the visibility of fitness short videos and the willingness to exercise with fitness influencers. The results from Elsotouhy et al. (29) indicated that the quality of information, system quality, and service quality on fitness platforms significantly impacted user engagement with fitness apps. Teng and Bao (30) discovered that interactions between individuals and information, as well as interactions among individuals, served as environmental stimuli that affected individuals’ internal states, thereby influencing their stickiness to fitness apps. This study suggests that when individuals use fitness apps, they often have certain goal-oriented needs. Users interact with others in the fitness app to obtain information and emotional supports from the app or other users. Thus, the social support can be regarded as an external stimulus. When individuals engage in upward and downward comparisons with other users, social comparison features of fitness apps help users perceive the “real presence” of other users more vividly, facilitating a precise assessment of their own fitness levels. Simultaneously, the heightened sense of competition enhances the enjoyment of fitness, increasing users’ interest in participating in fitness activities, thereby strengthening their continuous use intention of fitness apps (31). Consequently, this study employs the SOR model to explore how the two external stimuli of social support and social comparison within the fitness environment influence individuals’ organism, subsequently affecting their continuous use intention.

## 3 Research model and research hypotheses

**Figure 1:**
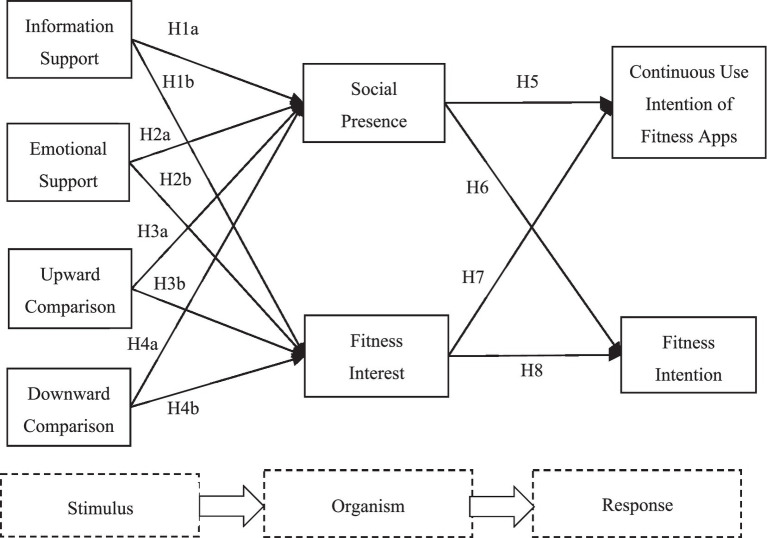
Research model.

This paper argues that the social support and social comparison features of fitness apps act as external stimuli that influence individuals’ organism states during usage, subsequently affecting their behavioral responses. Accordingly, this paper combines social support theory and social comparison theory to develop a research model based on the SOR theory, as shown in Figure 1.

### 3.1 Social support, social presence, and fitness interest

Social support theory has been utilized in the study of online and brand communities. For instance, Trepte et al. (32) discovered that online communities provided social support and a sense of competence to their users. Zhao et al. (33) classified social support into information support and emotional support, finding that social support positively affected users’ perceived benefits during online information searches. In this study, information support refers to the recommendations and suggestions provided by fitness apps that assist individuals in engaging in fitness activities more efficiently and safely. When individuals encounter challenges, they can quickly receive informational assistance from fitness apps or other users. Fitness apps integrate a variety of functions including AI coaching, intelligent pacing, dietary analysis, and personalized exercise plans. AI coaching delivers professional exercise demonstrations, interactive Q&A, image recognition, and voice-guided instructions, enhancing training safety and efficiency of users. Dietary analysis function automatically identifies food calories and nutritional components, aiding users in recording and analyzing their diets to achieve weight loss goals. Fitness apps can also generate personalized fitness plans based on user information, daily exercise records, and fitness goals, and monitor the implementation of users. These integrated functions provide users with information support, enhancing their fitness knowledge, improving fitness outcomes, and facilitating the achievement of fitness goals.

Literature indicates that in the context of online shopping, information support can moderate the effect of social presence on trust in online merchants (34). When using fitness apps, users genuinely perceive communication with others when they receive social support from other users, leading to feelings of warmth and closeness. On one hand, fitness apps provide individuals with more professional fitness information and guidance, helping them manage health issues and improve their fitness management levels. On the other hand, through comment interactions, users can view comments from other users, and these suggestions assist them in making better decisions. Particularly when users encounter difficulties and confusion, the advice provided by others helps them overcome challenges, making them feel that they are not alone and thus providing a sense of psychological companionship. It is evident that the information support provided by fitness apps enhances the social presence of users.

Fitness apps offer users a variety of fitness courses, personalized fitness plans, and professional online fitness guidance, all of which help alleviate fitness-related stress and enable users to engage in fitness activities more easily and enjoyably. The extensive social interaction features within the app enhance its appeal, encouraging users to continue using it. Therefore, it is evident that the information support function of fitness apps plays a significant role in enhancing users’ fitness interest. Consequently, we propose the following hypotheses:

*H1a*. Information support is significantly positively correlated with social presence.

*H1b*. Information support is significantly positively correlated with fitness interest.

Emotional support refers to the emotional and spiritual assistance received from fitness apps, such as care and attention. When individuals feel encouraged and supported, their confidence in interacting within the app is enhanced. Users can engage with other users within the fitness apps to receive emotional support, which helps motivate them to participate in fitness activities more enthusiastically. Previous research has indicated a positive correlation between emotional support and social presence. Emotional support helps reduce dissatisfaction and stress, assists individuals in overcoming challenges, thereby alleviating pressure and enhancing positive emotions (35). For instance, when users feel disappointed and frustrated during fitness process, other users can communicate with and comfort them. When users feel lonely during their fitness activities, other users can provide companionship (36). The emotional support helps users minimize emotional adversity, alter their negative perceptions toward fitness issues, and promote a positive understanding of fitness outcomes. Consequently, users can restore their confidence and positive attitude toward fitness and regain emotional stability when dealing with fitness-related issues. With the emotional support received, users experience feelings of being understood, comforted, and motivated, which in turn enhances their fitness interest. Conversely, when users encounter setbacks during their fitness journey and lack relevant emotional support, their enthusiasm and passion for fitness will decrease. Therefore, we propose the following hypothesis:

*H2a*. Emotional support is significantly positively correlated with social presence.

*H2b*. Emotional support is significantly positively correlated with fitness interest.

### 3.2 Social comparison, social presence, and fitness interest

Upward comparison refers to comparing oneself with individuals who perform better in a certain task, while downward comparison refers to comparing oneself with individuals who perform worse (37). Research indicates that, regarding fitness behaviors, individuals tend to compare themselves with other users in their social networks, and social comparison is associated with a significant increase in fitness activities (38). In particular, health interventions that utilize social comparison to create a competitive social environment can enhance the effectiveness of fitness activities.

Recently, fitness apps generally track users’ exercise records and rank their fitness performance against friends within the app. Features such as performance rankings enhance the importance of social comparison for fitness app users and should be examined as a behavior change mechanism within the fitness environment. While early research connected mobile fitness features (such as rankings) to the social comparison process, few studies have verified whether upward or downward comparisons are activated in this context. Recent studies have primarily focused on fitness posts shared on social platforms, confirming that social comparison can influence individuals’ exercise attitudes and fitness intentions. For example, Burke and Rains (17) found that both upward and downward comparisons increased concerns about weight when observing others’ exercise-related posts, with only downward comparisons showing a negative association with exercise attitudes. In the study of fitness posts on Instagram, Peng et al. (39) discovered that upward comparisons could motivate individuals to engage in physical exercise through self-enhancement, while no such effect was found for downward comparisons. Based on it, this study argues that the mechanism by which the social comparison features of fitness apps influence individuals’ organism states, thereby affecting fitness behaviors, requires further investigation. And it is essential to distinguish between different types of social comparisons.

In fitness apps, when individuals compare themselves to those who have better performance, they are more likely to regard these individuals as goals and believe that they can attain better fitness achievements in the future, thereby engaging in fitness activities more efficiently. Users are easily motivated, which can lead to positive emotions and an increase in fitness interest. Conversely, when individuals compare themselves to those who perform worse, users may experience feelings of shame and frustration. Engaging in downward comparison can lead to a decrease in fitness interest, as users may worry about deteriorating their performance to the level of the downward target, resulting in negative emotions (13). Therefore, we propose the following hypothesis:

*H3a*. Upward comparison is significantly positively correlated with social presence.

*H3b*. Upward comparison is significantly positively correlated with fitness interest.

*H4a*. Downward comparison is significantly negatively correlated with social presence.

*H4b*. Downward comparison is significantly negatively correlated with fitness interest.

### 3.3 Social presence, continuous use intention of fitness apps, and fitness intention

Social presence refers to the degree to which individuals feel the presence of others during communication, reflecting the sense of intimacy or direct experience that individuals develop toward the communication medium in interpersonal interactions (40). Social presence plays a crucial role in users’ psychology and behavior. When using fitness apps, users communicate with others, allowing them to compare their own fitness situations with other users. Through interaction, users can help bridge the social distance between them, enhancing their perception of others’ presence in fitness apps. As users experience a sense of authenticity and warmth from other users, they are more likely to engage in interpersonal interactions, perceive support and trust, thereby leading to continuous use of the fitness apps and an increased fitness intention. Existing literature has confirmed that social presence positively affects consumers’ online purchase intentions in social commerce (41). In related studies on online platforms, scholars have found that social presence not only affects users’ initial trust in the website but also influences their level of affection for the site and perceived usefulness (42). In online live streaming, social presence enhances consumers’ sense of identity, thereby increasing their purchase intentions (40). Another study indicates that social presence has a positive and significant impact on consumers’ trust and flow state, thereby triggering impulsive buying behavior (26). It is evident that social presence significantly influences users’ online purchase intentions, affection, trust, and sense of identity. This study applies the concept of social presence in the fitness context. When users perceive the presence of others, for instance, by interacting with other users through comments, obtaining necessary information, and receiving encouragement from others, their affection and trust for fitness apps will increase, which helps enhance their continuous use intention. Therefore, this study posits that the stronger the social presence perceived by users while using fitness apps, the more likely they are to increase their continuous use intention of fitness apps.

The higher the level of social presence that users experience while using fitness apps, the greater their motivation and intention to engage in fitness activities, such as exercise, diet, and monitoring. Previous studies have confirmed that social presence significantly impacts students’ satisfaction with online learning (43). In research on live streaming, scholars found that social presence positively influenced users’ willingness to watch live broadcasts (44). In this study, when users feel the presence and companionship of other users and receive social support from them, the sense of distance between users diminishes, and users’ intention to continue exercising increases. Therefore, we propose the following hypothesis:

*H5*. Social presence is significantly positively correlated with continuous use intention of fitness apps.

*H6*. Social presence is significantly positively correlated with fitness intention.

### 3.4 Fitness interest, continuous use intention of fitness apps, and fitness intention

Fitness interest refers to the degree of attention or interest that users have toward the outcomes of fitness behaviors (45). Interest is the first step in guiding users to engage in activities. Therefore, cultivating users’ interest in fitness behaviors is crucial, as this may lead them to adopt a positive attitude toward health behaviors and make better health-related choices. For instance, in this study, fitness apps can provide users with useful information support and positive feedback regarding fitness outcomes, thereby increasing their expectations of behavioral results and maintaining their interest in future fitness activities. Existing research indicates that fitness interest influences fitness behaviors (46, 47), and enhancing exercise interest will increase physical activity among middle school students, fostering a regular exercise lifestyle (48). In studies involving smartwatches, exercise interest is a significant criterion for segmenting smartwatch users’ exercise habits (49). In the fitness context, users’ fitness interest will evoke positive impressions of fitness apps and trigger users’ continuous use intention. Specifically, users’ fitness interest during the use of fitness apps helps to establish a deeper connection between the app and the user, enhancing user satisfaction and stickiness toward the fitness apps, thereby increasing the adoption rate.

From a hedonic perspective, as individuals cultivate an interest in using fitness apps, their enthusiasm and enjoyment for fitness activities also increase, thereby encouraging further participation in fitness (50). Since interest is a strong predictor of behavioral intention (51), users who develop interest, appreciation, and enjoyment for fitness activities while using fitness apps are more likely to engage in actual fitness activities. Because they believe they will derive greater utilitarian achievements, social value, and hedonic value from these activities (52). Therefore, we propose the following hypothesis:

*H7*. Fitness interest is significantly positively correlated with continuous use intention of fitness apps.

*H8*. Fitness interest is significantly positively correlated with fitness intention.

## 4 Research design and data analysis

### 4.1 Questionnaire design and variable measurement

This study employs the questionnaire survey method to collect data. The questionnaire is divided into three parts: the first part includes basic descriptive information about the questionnaire, providing relevant explanations about fitness apps. It guides respondents with the following prompt: “The mobile fitness application referred to in this questionnaire is an application that allows users to engage in fitness training through mobile devices, such as Keep, Joyrun, Mint Health, Xiaomi Sports, and Codoon. Please fill out this questionnaire based on your most recent experience using a fitness app.” The second part surveys the basic information of the respondents, including gender, age, education level and usage of fitness apps. The third part measures eight latent variables related to the social features of fitness apps, users’ organism and continuous use intention. To ensure the reliability and validity of the scales, the measurement items for the variables in this study are referenced from mature scales. Specifically, information support is based on the studies by Oh et al. (53) and Lin et al. (54), emotional support is based on the studies by Lin et al. (54) and Liang et al. (55). Upward and downward comparisons reference Kim’s (56) research and are adapted to fit the context of fitness apps. Social presence is based on the studies by Gao et al. (57) and Chen et al. (40), fitness interest is based on research of Jang et al. (45). Continuous use intention of fitness apps is based on the studies by Hsiao and Chiou (58) and Esteves et al. (19). And fitness intention is based on Bhattacherjee’s (59) research. Specific measurement items are shown in Table 1. Except for the statistical characteristics of the sample, all measurement items are assessed using a 7-point Likert scale, with 1 to 7 representing levers from “strongly disagree” to “strongly agree.” The questionnaire was sent to experts in the field of e-commerce for review. After multiple revisions based on expert feedback, the initial questionnaire was formed. The initial questionnaire was randomly sent online to 50 respondents for a pre-survey, and based on the results of the pre-survey, the content and wording of the items were revised to form the final questionnaire.

**Table 1:** Measurement items for each construct.

Constructs	Measurement items	References
Information support (IS)	IS1: The fitness app provides me with fitness information (e.g., how to effectively arrange exercise and diet) IS2: The fitness app offers strategies, suggestions, and advice for addressing fitness issues IS3: I can obtain a wealth of fitness-related information from the fitness app	Oh et al. (53), Lin et al. (54)
Emotional support (ES)	When I encounter fitness issues, fitness app users will ES1: Comfort and encourage me	Lin et al. (54), Liang et al. (55)
	ES2: Show concern for my mood and emotions	
	ES3: Provide me with emotional support	
Upward comparison (UC)	UC1: I believe it is important to compare myself with individuals who have better fitness achievements or higher fitness rankings than I do	Kim (56)
	UC2: I frequently compare myself with individuals who have better fitness achievements or higher fitness rankings than I do	
	UC3: I tend to compare myself with individuals whose fitness achievements are above average	
	UC4: I reflect on how competitive my fitness achievements are in comparison to those who excel	
Downward comparison (DC)	DC1: I believe it is important to compare myself with individuals who have worse fitness achievements or lower fitness rankings than I do	Kim (56)
	DC2: I often compare myself with individuals who have worse fitness achievements or lower fitness rankings than I do	
	DC3: I tend to compare myself with individuals whose fitness achievements are below average	
	DC4: I reflect on how competitive my fitness achievements are in comparison to those who perform poorly	
Social presence (SP)	SP1: There is a sense of human contact toward fitness app	Gao et al. (57), Chen et al. (40)
	SP2: There is a sense of human warmth toward fitness app	
	SP3: There is a sense of personness toward fitness app	
Fitness interest (FT)	FT1: I am interested in fitness activities, such as fitness training and running FT2: I have an interest in fitness-related information FT3: I am becoming increasingly interested in fitness	Jang et al. (45)
Continuous use intention of fitness apps (CU)	CU1: In the future, I will continue to use the fitness app CU2: In the future, I will use the fitness app frequently CU3: I will introduce the advantages of fitness app to others	Hsiao and Chiou (58), Esteves et al. (19)
Fitness intention (FI)	FI1: I am willing to make a genuine effort to maintain (or improve) my fitness ability FI2: I am willing to invest more time and energy to maintain (or enhance) my fitness level FI3: I am committed to engaging in fitness activities regularly	Bhattacherjee (59)

### 4.2 Data collection and sample description

The questionnaire was distributed and collected both online and offline using Questionnaire Star. Online distribution was conducted through WeChat, QQ, and email to relatives and friends. As for offline, it was distributed in classrooms by scanning QR codes for university students, and student survey teams were organized in densely populated areas such as shopping malls and cinemas to distribute the questionnaire to passersby via QR code scanning. A total of 500 questionnaires were distributed, with 378 returned. After excluding those that did not use any fitness apps, those with contradictory trap questions, those with completely identical answer options, and those with response times less than 1 min, 343 valid questionnaires remained. The descriptive statistics is presented in Table 2. Among the participants in the survey, 58.60% were male and 41.40% were female; the education level was primarily concentrated in Bachelor’s and Master’s degrees, accounting for 82.22% of the overall sample; the age group was mainly between 18 and 45, comprising 89.50% of the total sample; and 67.93% of the sample had over 1 year of experience using fitness apps.

**Table 2:** Descriptive statistics of the sample.

Item	Option range	Sample size	Percentage (%)	Item	Option range	Sample size	Percentage (%)
Gender	Male	201	58.60	Education level	High school or below	29	8.45
	Female	142	41.40		Associate degree	32	9.33
Age	Under 18	10	2.92		Bachelor’s degree	167	48.69
	18–24	94	27.41		Master’s degree or above	115	33.53
	25–35	126	36.73	Usage of fitness apps	Within 1 year	110	32.07
	36–45	87	25.36		1–3 years	143	41.69
	46–60	24	7.00		3–5 years	78	22.74
	60 and above	2	0.58		More than 5 years	12	3.50

### 4.3 Reliability and validity testing

SPSS 26.0 was utilized to assess the internal reliability of the research variable scales using Cronbach’s α. A Cronbach’s α value greater than 0.7 indicates good reliability, with higher values reflecting greater reliability (60). Typically, a CA coefficient value exceeding 0.6 suggests that the reliability level of the observed variables is acceptable (60). The Cronbach’s α coefficients for each research variable are presented in Table 3, where the coefficients for information support, emotional support, upward comparison, downward comparison, social presence, fitness interest, continuous use intention of fitness apps, and fitness intention all exceed 0.8, indicating that the scale possesses high internal consistency and can reliably measure the latent variables.

**Table 3:** Reliability and validity results of each construct.

Constructs	Items	Factor loadings	Cronbach’s α	CR	AVE
Information support	IS1	0.832	0.820	0.821	0.605
	IS2	0.777			
	IS3	0.721			
Emotional support	ES1	0.808	0.850	0.85	0.655
	ES2	0.825			
	ES3	0.794			
Upward comparison	UC1	0.792	0.877	0.877	0.64
	UC2	0.793			
	UC3	0.798			
	UC4	0.817			
Downward comparison	DC1	0.862	0.927	0.928	0.763
	DC2	0.874			
	DC3	0.875			
	DC4	0.882			
Social presence	SP1	0.826	0.881	0.88	0.71
	SP2	0.856			
	SP3	0.845			
Fitness interest	FT1	0.874	0.916	0.915	0.782
	FT2	0.881			
	FT3	0.898			
Continuous use intention of fitness apps	CU1	0.873	0.921	0.922	0.796
	CU2	0.900			
	CU3	0.904			
Fitness intention	FI1	0.879	0.869	0.87	0.691
	FI2	0.800			
	FI3	0.812			

To validate the reliability and validity of the measurement model, confirmatory factor analysis was conducted using AMOS 26.0, with the results presented in Table 3. The findings indicate that the standardized factor loadings for each observed variable exceed 0.7. Calculations reveal that the composite reliability (CR) for each factor is greater than 0.8, indicating strong reliability for each factor (61). The average variance extracted (AVE) for latent variables is above 0.6, demonstrating good convergent validity (61). And it is observed that the square roots of AVE are always greater than the absolute correlations between the constructs. AVE ranges from 0.778 to 0.892 (see Table 4). It indicates that all the constructs report acceptable validity (61).

**Table 4:** Correlations between variables and square roots of average variance extracted.

Constructs	1	2	3	4	5	6	7	8
1. Information support	0.778							
2. Emotional support	0.305	0.809						
3. Upward comparison	0.241	0.355	0.800					
4. Downward comparison	0.278	0.304	0.292	0.873				
5. Social presence	0.280	0.334	0.315	0.340	0.842			
6. Fitness interest	0.327	0.369	0.336	0.306	0.304	0.884		
7. Continuous use intention of fitness apps	0.250	0.311	0.220	0.301	0.276	0.332	0.892	
8. Fitness intention	0.312	0.338	0.283	0.306	0.225	0.368	0.305	0.831

### 4.4 Hypothesis testing

The path coefficients and validation results of the model are presented in Table 5, where hypothesis H4b was not supported, while the other hypotheses were supported.

**Table 5:** Path coefficients and validation results of the model.

Path model	Path coefficients	*T*-value	Significance	Results
H1a Information support → Social presence	0.160	2.456	*	Supported
H1b Information support → Fitness interest	0.297	4.539	***	Supported
H2a Emotional support → Social presence	0.218	2.988	**	Supported
H2b Emotional support → Fitness interest	0.213	2.943	**	Supported
H3a Upward comparison → Social presence	0.176	2.566	**	Supported
H3b Upward comparison → Fitness interest	0.185	2.745	**	Supported
H4a Downward comparison → Social presence	−0.261	−4.359	***	Supported
H4b Downward comparison → Fitness interest	−0.098	−1.643	ns	Not supported
H5 Social presence → Continuous use intention of fitness apps	0.230	3.812	***	Supported
H6 Social presence → Fitness intention	0.183	3.051	**	Supported
H7 Fitness interest → Continuous use intention of fitness apps	0.351	5.849	***	Supported
H8 Fitness interest → Fitness intention	0.451	7.419	***	Supported

*** indicates a significance level of 0.001, ** indicates a significance level of 0.01, * indicates a significance level of 0.05, and ns indicates not significant.

### 4.5 Mediation effect test

This paper employs the Bootstrap method to test the mediating effects of social presence and fitness interest. The Bootstrap method is a commonly used resampling technique in mediation analysis, which estimates the indirect effects of mediating variables and the characteristics of the sampling distribution through repeated random sampling, providing confidence intervals for the indirect effects. When the confidence interval does not include 0, the effect is considered significant. Using AMOS 26.0, the Bootstrap random sampling was set to 5,000 iterations, and the mediation effects were tested at a 95% confidence interval. A parallel mediation model was employed for testing, and the results are presented in Table 6. Except for the mediating effects of fitness interest in the downward comparison with continuous use intention of fitness apps and downward comparison with fitness intention, which were not significant, all other mediating effects were significant and indicated partial mediation.

(1) Social presence mediates the positive relationship between information support and continuous use intention, as well as between information support and fitness intention. Social presence mediates the positive relationship between emotional support and continuous use intention, as well as between emotional support and fitness intention. Social presence mediates the positive relationship between upward comparison and continuous use intention, as well as between upward comparison and fitness intention. Social presence mediates the negative relationship between downward comparison and continuous use intention, as well as between downward comparison and fitness intention.
(2) Fitness interest mediates the positive relationship between information support and continuous use intention, as well as between information support and fitness intention. Fitness interest mediates the positive relationship between emotional support and continuous use intention, as well as between emotional support and fitness intention. Fitness interest mediates the positive relationship between upward comparison and continuous use intention, as well as between upward comparison and fitness intention.

**Table 6:** Bootstrap mediation effect test results.

Pathway	Coefficient	Boot LLCI	Boot ULCI	*p*	Results
Information support → Social presence → Continuous use intention	0.068	0.028	0.121	0.003	Supported
Information support → Fitness interest → Continuous use intention	0.134	0.072	0.217	0.004	Supported
Information support → Social presence → Fitness intention	0.034	0.004	0.079	0.074	Supported
Information support → Fitness interest → Fitness intention	0.130	0.081	0.197	0.001	Supported
Emotional support → Social presence → Continuous use intention	0.064	0.017	0.122	0.014	Supported
Emotional support → Fitness interest → Continuous use intention	0.125	0.072	0.207	0.005	Supported
Emotional support → Social presence → Fitness intention	0.025	0.016	0.079	0.033	Supported
Emotional support → Fitness interest → Fitness intention	0.126	0.074	0.181	0.013	Supported
Upward comparison → Social presence → Continuous use intention	0.081	0.028	0.148	0.007	Supported
Upward comparison → Fitness interest → Continuous use intention	0.144	0.083	0.225	0.007	Supported
Upward comparison → Social presence → Fitness intention	0.043	0.004	0.097	0.072	Supported
Upward comparison → Fitness interest → Fitness intention	0.147	0.099	0.204	0.001	Supported
Downward comparison → Social presence → Continuous use intention	−0.056	−0.107	−0.013	0.013	Supported
Downward comparison → Fitness interest → Continuous use intention	−0.098	−0.170	0.059	0.103	Not supported
Downward comparison → Social presence → Fitness intention	−0.105	−0.150	−0.073	0.009	Supported
Downward comparison → Fitness interest → Fitness intention	−0.028	−0.076	0.011	0.157	Not supported

## 5 Discussion

This study employs the SOR model, utilizing the social support and social comparison features of fitness apps as stimulus variables, and social presence and fitness interest as organism variables, to validate their impact on users’ continuous use intention and fitness intention, yielding the following research results.

### 5.1 The impact of social features in fitness apps

(1) Information support positively influences users’ social presence and fitness interest. Furthermore, information support has a significant positive effect on users’ continuous use intention and fitness intention through the mediation of social presence and fitness interest. It indicates that the information support obtained from fitness apps not only allows users to perceive the presence of other users but also enhances their fitness interest. Together, these factors promote users to continue using fitness apps and increase their fitness intention. This finding aligns with the results of Sun and Jiang (12), which suggest that information support positively influences fitness intention through the mediation of fitness attitude. Thus, it is evident that the information support feature of fitness apps enables users to understand the benefits of fitness, which helps cultivate a positive interest in fitness and enhances their fitness intention.
(2) Emotional support positively influences users’ social presence and fitness interest. Furthermore, emotional support has a substantial positive impact on users’ continuous use intention and fitness intention through the mediation of social presence and fitness interest. Emotional support positively affects both social presence and fitness interest, with similar path coefficients, indicating that emotional support obtained from the fitness apps helps users perceive the care of other users, alleviating stress and fostering positive emotions, thereby promoting users’ continuous use. Additionally, emotional support encourages users to develop an interest in fitness activities, such as running and fitness training with other users. As a social networking platform, fitness apps possess entertainment attributes. Thus, ensuring that users feel pleasure and receive emotional support during their usage is crucial, which also contributes to enhancing users’ continuous use of the fitness apps. Because our data are cross-sectional, we assume these relationships are causal (e.g., support leads to interest), but future longitudinal studies are needed to confirm causality.
(3) Upward comparison positively influences users’ social presence and fitness interest. Furthermore, upward comparison has a significant positive effect on users’ continuous use intention and fitness intention through the mediation of social presence and fitness interest. This finding contrasts with the results of Nunnally and Bernstein Tong et al. (62), which suggest that comparing oneself to higher standards is discouraging. In contrast, they prefer to compare themselves with those who perform worse to gain confidence. However, the results of this study indicate that comparing oneself to users with better performances while using fitness apps helps create a competitive environment, motivating users to achieve better results and thereby enhancing the effectiveness of fitness activities. Additionally, through the mediating roles of social presence and fitness interest, upward comparison is associated with an increase in continuous use intention and fitness intention of fitness apps. This study contributes to the literature on fitness apps by exploring the positive effects of upward comparison.
(4) Downward comparison negatively affects users’ social presence, while its impact on fitness interest is not significant. Furthermore, downward comparison negatively influences users’ continuous use intention and fitness intention through the mediation of social presence. The lack of significant impact of downward comparison on fitness interest indicates that comparing oneself to others with inferior fitness achievements does not lead to a stronger fitness interest among users. Since, when users engage in downward comparison, even if their achievements surpass other users, they may not experience feelings of happiness or excitement. Instead, they might perceive it as a normal occurrence, potentially leading to feelings of shame and frustration. Consequently, the impact of downward comparison on fitness interest is not significant. Downward comparison negatively affects users’ social presence. The reason is that engaging in downward comparison hinders users from developing a sense of closeness and warmth toward other users within the fitness apps (40). It’s difficult to impact users’ conformity psychology and behavior indirectly through identity recognition and normative influence when comparing them with users with inferior fitness achievements. On the other hand, users may develop feelings of complacency and lose the motivation to achieve better results. Thus, downward comparison also fails to enhance users’ fitness intention. Previous studies have indicated that in situations such as depression or dieting behaviors, downward comparison plays a more dominant role and produces negative health effects (63). The results of this study suggest that in the fitness context, downward comparison negatively impacts users’ social presence, which in turn negatively affects their continuous use intention and fitness intention.

### 5.2 The impact of organism

(1) Social presence positively influences users’ continuous use intention and fitness intention of fitness apps, with both path coefficients being highly significant. Previous studies have shown that social presence positively affects viewers’ willingness to watch live streaming (44) and their online shopping intention (34). The results of this study indicate that users can perceive the presence of other users while using fitness apps, which helps cultivate a sense of interaction, support, and trust. These psychological factors can enhance users’ continuous use intention and fitness intention. Therefore, fitness app managers should provide social features that enable users to feel a connection with others during their fitness journey, experiencing warmth, companionship, and interaction, thereby promoting continuous use.
(2) Fitness interest positively influences users’ continuous use intention and fitness intention. Fitness interest emerged as a particularly strong predictor of fitness intention, more than social presence, suggesting that fostering genuine interest might yield the greatest returns in sustained fitness commitments (45). Therefore, fitness app practitioners should design features that align with users’ interests to enhance the interactivity of the app and provide more diverse experiences along with professional information support. Such as developing virtual avatars and question-answering functions to stimulate users’ fitness interest and thereby promote their continuous use. And if fitness apps incorporate these supportive and comparative features effectively, through the mediation of social presence and fitness interest, they could become more reliable tools for public health promotion by keeping people engaged in physical activity regimes. Thereby potentially improving population health outcomes.

## 6 Conclusion

### 6.1 Research conclusions

This study employs fitness apps as the research context and constructs a research model based on SOR theory to investigate the impact of social features of fitness apps on users’ continuous use intention and fitness intention. Data were collected through a questionnaire. The empirical findings indicate that the social support and social comparison features of fitness apps exert varying degrees of stimulating effects on users’ social presence and fitness interest, thereby positively influencing users’ continuous use intention and fitness intention.

### 6.2 Research significance

#### 6.2.1 Theoretical significance

This study presents the following theoretical significance. First, we examine the social features and social value of fitness apps by categorizing these features into social support and social comparison. The findings confirm that social support and social comparison, as external factors, significantly influence users’ organism, as well as their behavioral intentions. It enriches the research content on fitness apps and expands the application scope of SOR theory, thereby contributing to the in-depth study of fitness apps. Second, this study innovatively defines users’ organism variables as social presence and fitness interest. And it verifies the mediating roles of social presence and fitness interest between stimuli and responses, enriching the research content on organism. Third, this study categorizes user behavioral responses into continuous use intention and fitness intention, validating the influence pathways of fitness app social features on these two behavioral intentions. Thereby enriching the research content on user behavior in the fitness domain.

#### 6.2.2 Practical significance

The findings of this study help fitness platform managers to develop more social features and realize the commercial value of fitness apps. The results facilitate users’ continuous use of fitness apps, promote positive fitness activities, and thus help users realize their health value. Meanwhile, the continuous use of fitness apps helps to create a positive fitness atmosphere throughout society. Especially, in terms of information support, app practitioners should provide information retrieval services for users to search for the information they need and receive appropriate guidance. Regarding emotional support, managers should enhance the social features of fitness apps to foster supportive online communities, promoting the well-being of users. In terms of social comparison, practitioners should regularly organize activities and establish reward mechanisms to motivate more users to participate and achieve better results. Furthermore, managers should promote fitness apps through social media to attract more people to participate in online fitness activities.

### 6.3 Research limitations and future directions

Due to objective constraints, this study has certain limitations: (1) This paper primarily examines two prominent social features in fitness apps, which presents a limitation. There is a plenty of social features of fitness apps, and future research can further incorporate additional factors to conduct a more systematic investigation into users’ behavioral intentions. (2) The social features of fitness apps can influence users’ organism and responses. The mechanisms underlying these influences, as well as the potential existence of other mediating and moderating variables, can be further explored in future research.

## Data Availability

The raw data supporting the conclusions of this article will be made available by the authors, without undue reservation.
